# Co-crystal structure of *Helicobacter pylori* biotin protein ligase with biotinyl-5-ATP

**DOI:** 10.1107/S2053230X24012056

**Published:** 2025-01-01

**Authors:** Jesuferanmi P. Ayanlade, Dylan E. Davis, Sandhya Subramanian, David M. Dranow, Donald D. Lorimer, Brad Hammerson, Peter J. Myler, Oluwatoyin A. Asojo

**Affiliations:** a Dartmouth Cancer Center, One Medical Center Drive, Lebanon, NH03756, USA; b College of Arts and Science Dartmouth College Hanover NH03755 USA; c Center for Global Infectious Disease Research Seattle Children’s Research Institute 307 Westlake Avenue North, Suite 500 Seattle WA98109 USA; d Seattle Structural Genomics Center for Infectious Diseases, Seattle, Washington, USA; e UCB BioSciences, Bainbridge Island, WA98110, USA; University of York, United Kingdom

**Keywords:** undergraduate education and training, SSGCID, infectious diseases, cancer, gastric ulcers

## Abstract

*Helicobacter pylori*, a type 1 carcinogen that causes human gastric ulcers and cancer, is a priority target of the Seattle Structural Genomics Center for Infectious Disease (SSGCID). These efforts include determining the structures of potential *H. pylori* therapeutic targets. Here, the purification, crystallization and X-ray structure of one such target, *H. pylori* biotin protein ligase (*Hp*BPL), are reported. *Hp*BPL catalyzes the activation of various biotin-dependent metabolic pathways, including fatty-acid synthesis, gluconeogenesis and amino-acid catabolism, and may facilitate the survival of *H. pylori* in the high-pH gastric mucosa. *Hp*BPL is a prototypical bacterial biotin protein ligase, despite having less than 35% sequence identity to any reported structure in the Protein Data Bank. A biotinyl-5-ATP molecule sits in a well conserved cavity. *Hp*BPL shares extensive tertiary-structural similarity with *Mycobacterium tuberculosis* biotin protein ligase (*Mt*BPL), despite having less than 22% sequence identity. The active site of *Hp*BPL is very similar to that of *Mt*BPL and has the necessary residues to bind inhibitors developed for *Mt*BPL.

## Abstract 2

Recombinant N-terminally histidine-tagged *H. pylori* biotin protein ligase was purified and its 2.25 Å resolution co-crystal structure with biotinyl-5-ATP is reported.

## 1. Introduction

Over half of the human population is infected with *Helicobacter pylori*, a spiral-shaped, flagellated, Gram-negative bacterium that is highly adapted for human colonization (Warren & Marshall, 1983 ▸; Malfertheiner *et al.*, 2023 ▸; Moss *et al.*, 2023 ▸). The presence of *H. pylori* increases the risk of noncardiac gastric adenocarcinoma, gastric lymphoma and peptic ulcer (Malfertheiner *et al.*, 2023 ▸; Moss *et al.*, 2023 ▸; Cover & Blaser, 2009 ▸). *H. pylori* was classified as a type 1 carcinogen in 1994 by the International Agency for Research on Cancer (Ahn & Lee, 2015 ▸; Malfertheiner *et al.*, 2023 ▸). The unique metabolic adaptations of *H. pylori* that support persistence in the harsh gastric mucosa include utilizing molecular hydrogen (H_2_) as an energy source, driving a chemolithoautotrophic growth mode (Kuhns *et al.*, 2016 ▸). This growth mode allows *H. pylori* to achieve higher growth yields and increased carbon fixation from bicarbonate under hydrogen-rich conditions such as in the gastric mucosa (Benoit *et al.*, 2020 ▸). Furthermore, increasing antimicrobial resistance of *H. pylori* has been reported (Elbehiry *et al.*, 2023 ▸). *H. pylori* is a priority target of the Seattle Structural Genomics Center for Infectious Disease (SSGCID). These efforts include structural studies of *H. pylori* proteins for rational drug discovery or repurposing. Here, we present structural studies on one of these proteins, *H. pylori* biotin protein ligase (*Hp*BPL), which catalyzes the transfer of biotin to biotin-accepting proteins. *Hp*BPL is vital for the structural integrity of the bacterial cell wall, and the *Mycobacterium tuberculosis* homolog has been investigated as a drug target (Duckworth *et al.*, 2011 ▸; Gupta *et al.*, 2010 ▸). *Hp*BPL is required for essential metabolic pathways, including fatty-acid synthesis, gluconeogenesis and amino-acid catabolism (Burns *et al.*, 1995 ▸). *Hp*BPL does not share any appreciable sequence identity with human proteins, making it an attractive target for drug discovery. Here, we report the production, crystallization and 2.25 Å resolution structure of *Hp*BPL.

## 2. Materials and methods

### 2.1. Macromolecule production

*Hp*BPL was cloned, expressed and purified as described previously (Stacy *et al.*, 2011 ▸; Serbzhinskiy *et al.*, 2015 ▸; Rodríguez-Hernández *et al.*, 2023 ▸). The full-length gene for biotin acetyl coenzyme A carboxylase synthetase from *H. pylori* G27 (UniProt B5Z8D8) encoding amino acids 1–212 was PCR-amplified from genomic DNA using the primers shown in Table 1 ▸. The gene was cloned into the expression vector BG1861 to generate plasmid DNA, which was transformed into chemically competent *Escherichia coli* BL21(DE3) Rosetta cells. The plasmid containing His-*Hp*BPL was tested for expression and 2 l of culture was grown using auto-induction medium (Studier, 2005 ▸) in a LEX Bioreactor (Epiphyte Three) as described previously (Serbzhinskiy *et al.*, 2015 ▸). The expression clone is available for request online at https://www.ssgcid.org/available-materials/expression-clones/.

*Hp*BPL was purified using a previously described two-step protocol consisting of an immobilized metal (Ni^2+^) affinity chromatography (IMAC) step followed by size-exclusion chromatography (SEC) on an ÄKTApurifier 10 (GE Healthcare) using automated IMAC and SEC programs (Serbzhinskiy *et al.*, 2015 ▸). Briefly, thawed bacterial pellets (25 g) were lysed by sonication in 200 ml lysis buffer [25 m*M* HEPES pH 7.0, 500 m*M* NaCl, 5%(*v*/*v*) glycerol, 0.5%(*w*/*v*) CHAPS, 30 m*M* imidazole, 10 m*M* MgCl_2_, 400 µg ml^−1^ lysozyme, 3 U ml^−1^ Benzonase]. After sonication, nucleic acids were degraded by incubation with 20 µl (25 U ml^−1^) Benzonase with mixing for 45 min at room temperature. The lysate was clarified by centrifugation at 10 000 rev min^−1^ for 1 h using a Sorvall centrifuge (Thermo Scientific). The clarified supernatant was then passed over an Ni–NTA HisTrap FF 5 ml column (GE Healthcare) which had been pre-equilibrated with wash buffer [25 m*M* HEPES pH 7.0, 500 m*M* NaCl, 5%(*v*/*v*) glycerol, 30 m*M* imidazole pH 7.0]. The column was washed with 20 column volumes (CV) of wash buffer and eluted with elution buffer [20 m*M* HEPES pH 7.0, 500 m*M* NaCl, 5%(*v*/*v*) glycerol, 500 m*M* imidazole pH 7.0] over a 7 CV linear gradient. The peak fractions were pooled and concentrated to 5 ml for size-exclusion chromatography (SEC). For SEC, the 5 ml protein sample was loaded onto a Superdex 75 26/60 column (GE Biosciences) attached to an ÄKTAprime plus FPLC system (GE Biosciences) that had been pre-equilibrated with SEC buffer [20 m*M* HEPES, pH 7.0, 300 m*M* NaCl, 5%(*v*/*v*) glycerol, 1 m*M* TCEP]. The column was washed with 100 ml of SEC buffer before fractions were collected at 1.5 ml min^−1^ using an additional 180 ml. The peak fractions were collected and assessed for purity by SDS–PAGE on a 4–20% Protein Gel (Invitrogen) and visualized by Coomassie staining with InstantBlue colloidal stain (Expedeon, San Diego, California, USA). *Hp*BPL eluted as a single, symmetrical, monodisperse peak accounting for >90% of the protein product at a molecular mass of ∼20 kDa, suggesting purification as a monomer (monomer expected molecular weight 25 kDa). The peak fraction was pooled and concentrated to ∼62.8 mg ml^−1^ using an Amicon purification system (Millipore). 110 µl aliquots were flash-frozen in liquid nitrogen and stored at −80°C until use. Recombinant *Hp*BPL is available for request online at https://targetstatus.ssgcid.org/Target/HepyC.19466.

### 2.2. Crystallization

His-tagged *Hp*BPL crystallized at 290 K using sitting-drop vapor diffusion directly from a JCSG+ screen condition (Table 2 ▸). *Hp*BPL at 62.8 mg ml^−1^ in SEC buffer was mixed with MgCl_2_, ATP and biotin, and the mixture was incubated at 25°C for 10 min to generate the protein–ligand mixture (23.6 mg ml^−1^*Hp*BPL with 6 m*M* MgCl_2_, 6 m*M* ATP and 6 m*M* biotin). 0.4 µl of the protein–ligand mixture was mixed with an equal volume of the precipitant solution in the well of a Rigaku Reagents XJR sitting-drop vapor-diffusion tray. 80 µl precipitant solution (JCSG+ condition B1; 0.1 *M* sodium citrate tribasic/citric acid pH 4.0, 0.8 *M* ammonium sulfate) was present in the reservoir (Table 2 ▸). Before data collection, the crystals were harvested and cryoprotected with 25%(*v*/*v*) ethylene glycol (Table 2 ▸).

### 2.3. Data collection and processing

Diffraction data were collected at 100 K on Advanced Photon Source (APS) beamline 21-ID-F at Argonne National Laboratory (Table 3 ▸). The data were integrated with *XDS* and reduced with *XSCALE* (Kabsch, 2010 ▸). Raw X-ray diffraction images were stored at the Integrated Resource for Reproducibility in Macromolecular Crystallo­graphy at https://www.proteindiffraction.org.

### 2.4. Structure solution and refinement

**Figure 1:**
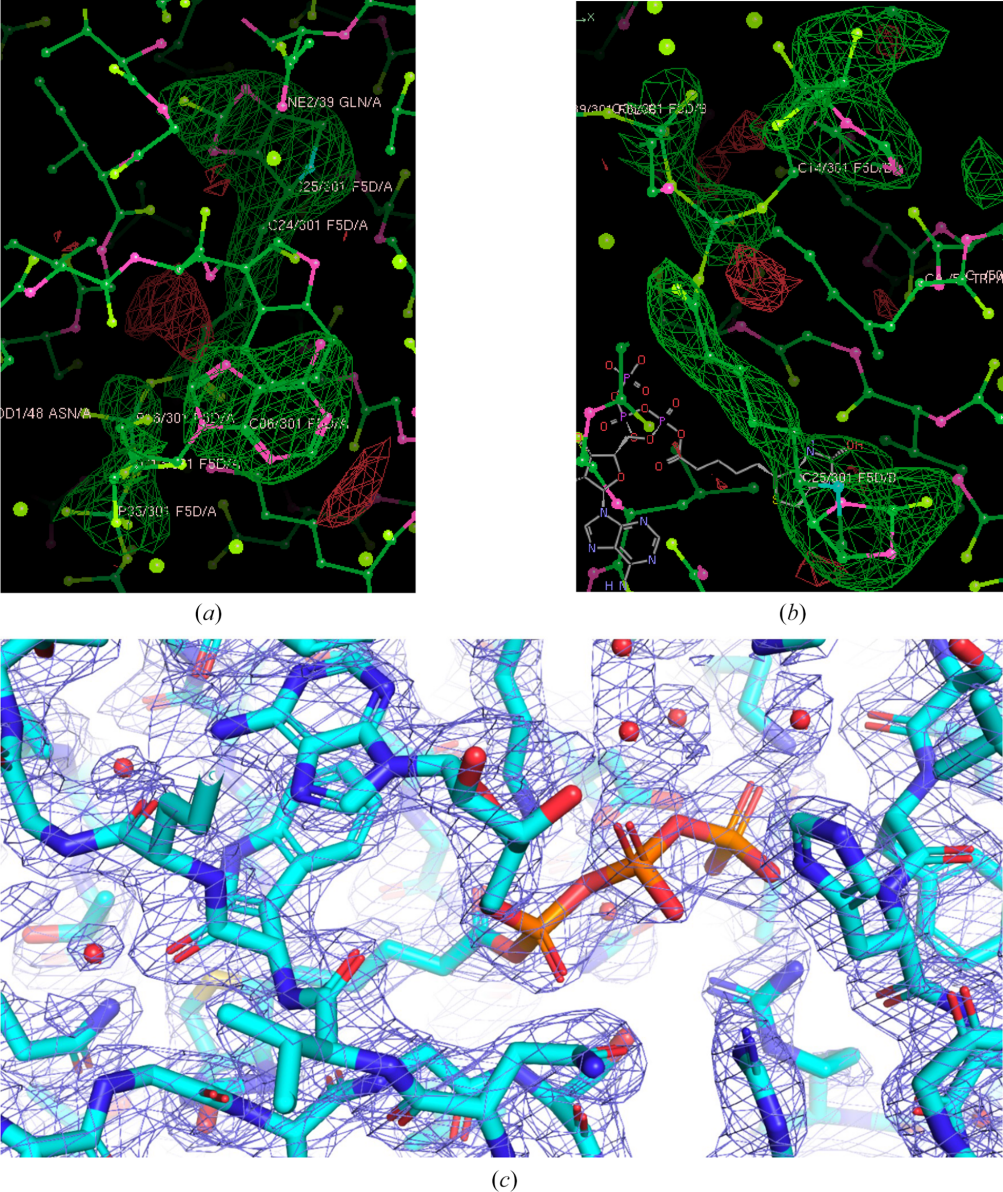
*Hp*BPL electron-density maps. The biotinyl-5-ATP from (*a*) monomer *A* and (*b*) monomer *B* fits into initial 3σ omit (*F*_o_ − *F*_c_) electron-density maps (green mesh). (*c*) The 1.2σ 2*F*_o_ − *F*_c_ electron-density map of *Hp*BPL is shown as a blue mesh.

The structure of *Hp*BPL was determined by molecular replacement using *Phaser* (McCoy *et al.*, 2007 ▸) from the *CCP*4 suite of programs (Collaborative Computational Project, Number 4, 1994 ▸; Krissinel *et al.*, 2004 ▸; Winn *et al.*, 2011 ▸; Agirre *et al.*, 2023 ▸) with PDB entry 3l1a (Gupta *et al.*, 2010 ▸) as the search model. The structure was refined using *Phenix* (Liebschner *et al.*, 2019 ▸). The omit *F*_o_ − *F*_c_ electron-density map for the biotinyl-5-ATP is well ordered (Fig. 1 ▸*a*). The model was built into high-quality 2*F*_o_ − *F*_c_ electron density (Fig. 1 ▸*b*). The structure quality was checked using *MolProbity* (Williams *et al.*, 2018 ▸). Data-reduction and refinement statistics are shown in Table 4 ▸. Coordinate and structure factors have been deposited in the Worldwide PDB (wwPDB) as entry 6ck0.

## 3. Results and discussion

Size-exclusion chromatography data suggest that *Hp*BPL assembles as a monodisperse monomer in solution with a molecular weight of ∼20 kDa, close to the theoretical mass of 25 kDa. Analysis with the *Protein Interfaces, Surfaces and Assembly* (*PISA*) service at the European Bioinformatics Institute (https://www.ebi.ac.uk/pdbe/prot_int/pistart.html) agrees with the SEC data that *Hp*BPL is indeed a biological monomer (Krissinel, 2015 ▸). Recombinant *Hp*BPL is catalytically active and generates biotinyl-5-ATP upon incubation with MgCl_2_, ATP and biotin, which is observed in the active site (Fig. 1 ▸).

**Figure 2:**
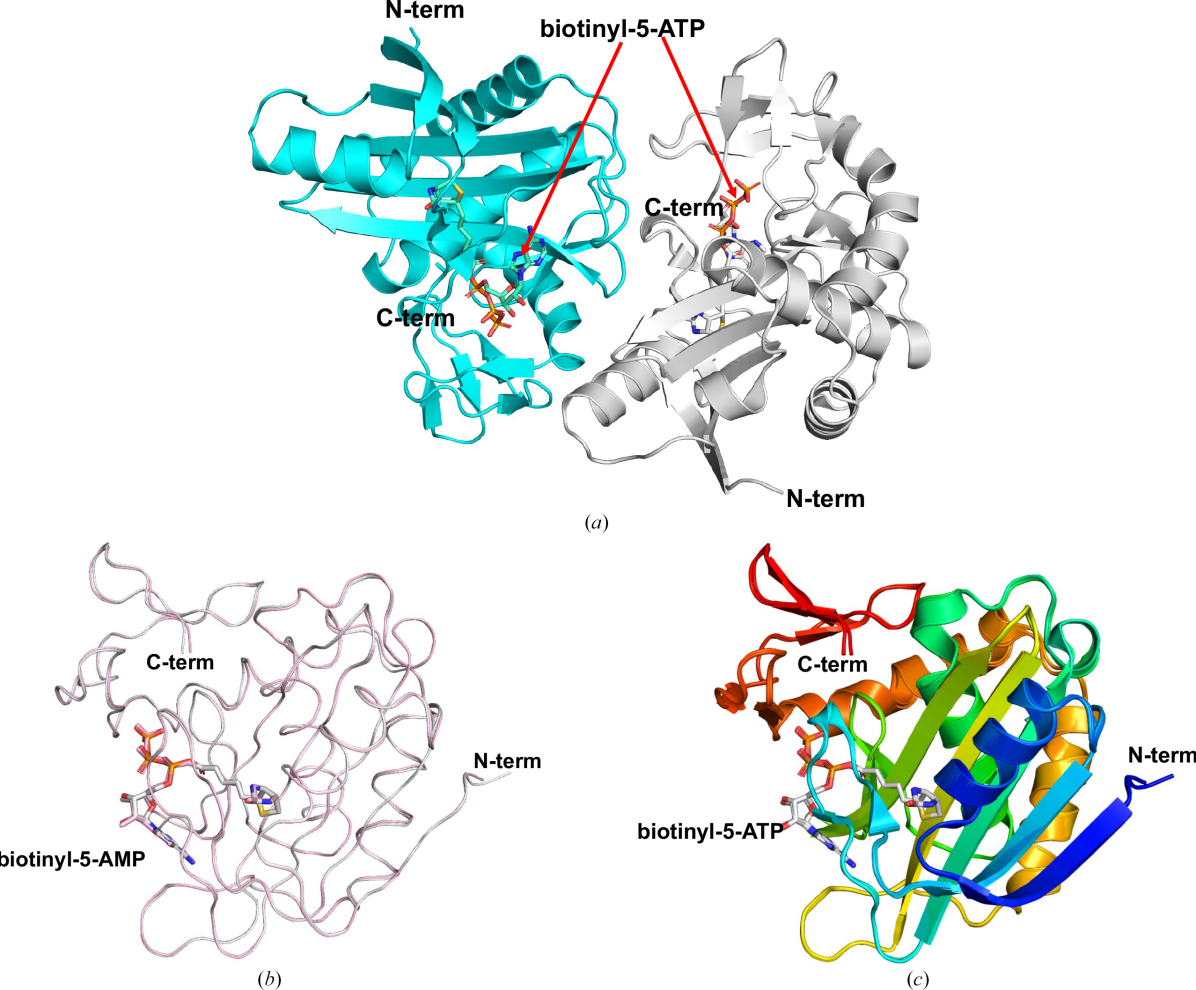
Overall structure of *Hp*BPL. (*a*) *Hp*BPL dimer. (*b*) Ribbon diagrams of superposed *Hp*BPL monomers reveal conserved topology; one monomer is gray and the other is pink. (*c*) Cartoon of *Hp*BPL colored in rainbow from blue at the N-terminus to red at the C-terminus.

The structure was refined in the triclinic space group *P*1 with two monomers in the asymmetric unit (Fig. 2 ▸*a*). Both monomers are similar, with an r.m.s.d. of 0.20 Å for all C^α^ atoms (Fig. 2 ▸*b*). Each monomer contains a biotinyl-5-ATP molecule in the central catalytic cavity (Figs. 1 ▸, 2 ▸ and 3 ▸). Both monomers include the following secondary structures: 34.4% strands, 22.5% α-helix and 6.7% 3_10_-helix. The 14 β-strands assemble as four β-sheets consisting of one seven-stranded mixed sheet, two two-stranded antiparallel sheets and a three-stranded antiparallel sheet (Fig. 2 ▸*c*). *Hp*BPL has eight helices, one β–α–β motif, four helix–helix interactions and 17 β-turns.

**Figure 3:**
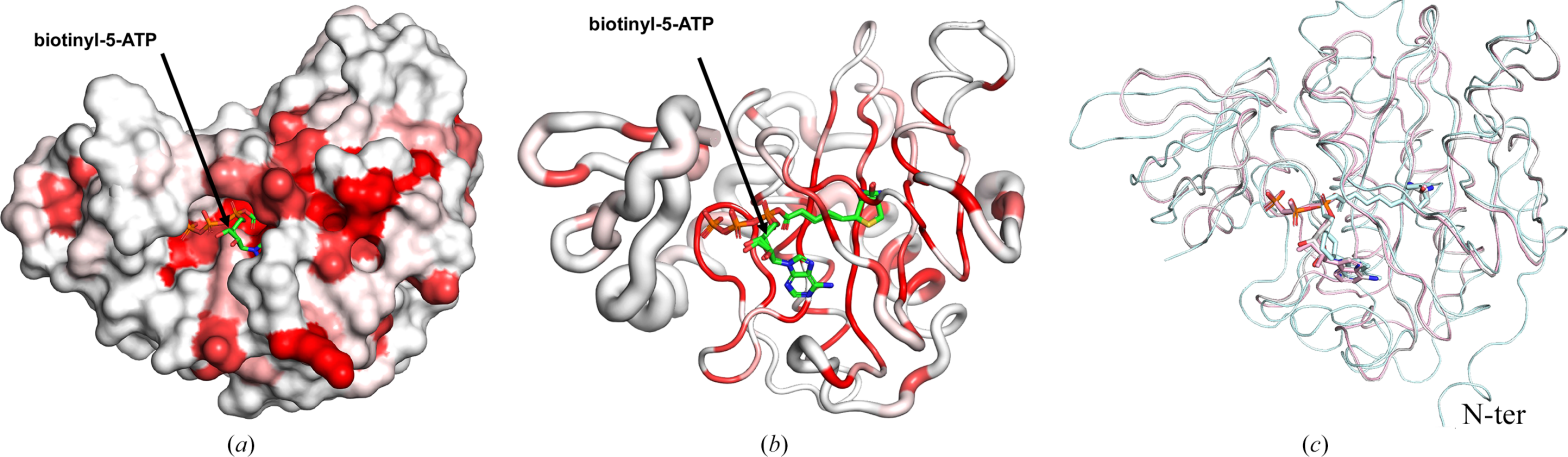
(*a*) The solvent-accessible surface area of *Hp*BPL is colored by sequence conservation, with red indicating identical residues. (*b*) Ribbon diagram calculated by *ENDScript*. The circumference of the ribbon (sausage) represents relative structural conservation compared with other BPL structures. Thinner ribbons represent more conserved regions. In comparison, thicker ribbons represent less conserved regions and the ribbon is colored by sequence conservation, with red indicating identical residues. (c) Alignment of *Hp*BPL and *Mt*BPL. The PDB entries of the protein structures used for this alignment are indicated in Supplementary Fig. S1.

*ENDScript* (Gouet *et al.*, 2003 ▸; Robert & Gouet, 2014 ▸) analysis reveals that *Hp*BPL has a prototypical bacterial biotin protein ligase topology (Supplementary Fig. S1). This is consistent with its InterPro classification as a member of the biotin–acetyl-CoA-carboxylase ligase (IPR004408) family and as a biotin protein ligase/lipoate protein ligase (BPL/LPL) catalytic domain-containing protein. Additionally, residues near biotinyl-5-ATP in the active sites of bacterial BPLs are well conserved, as indicated by the red color in the sausage and surface plots (Figs. 3 ▸*a* and 3 ▸*b*). Furthermore, the thinness of the sausage plot reveals the well conserved tertiary structure of biotinyl-5-ATP-binding regions among bacterial BPLs (Fig. 3 ▸*a*).

**Figure 4:**
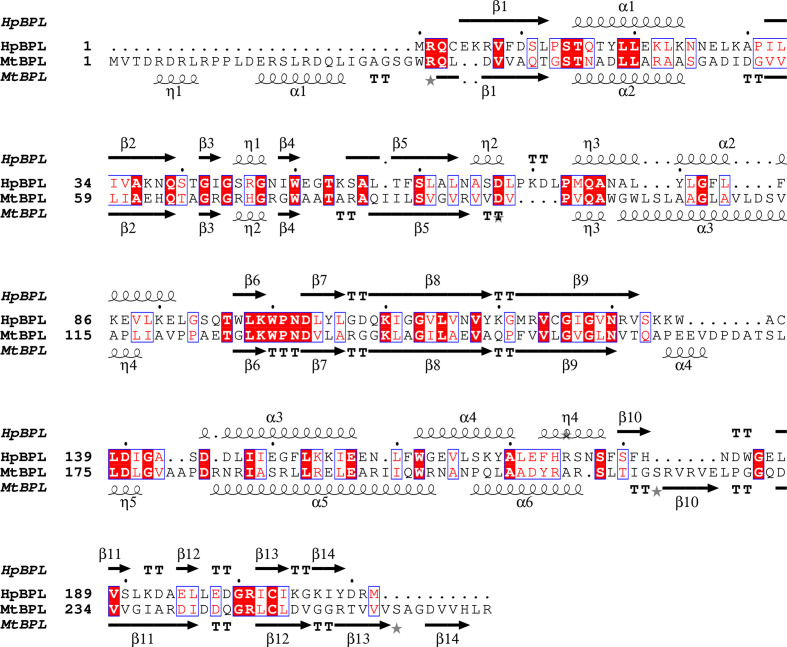
Structural and primary-sequence alignment of *Hp*BPL (PDB entry 6ck0) and *Mt*BPL (PDB entry 4xu1). The secondary-structure elements are as follows: α-helices are shown as large coils, 3_10_-helices are shown as small coils labeled η, β-strands are shown as arrows labeled β and β-turns are labeled TT. The identical residues are shown on a red background, with conserved residues in red and conserved regions in blue boxes. Fig. 4 ▸ was generated using *ESPript* 3.0 (Gouet *et al.*, 1999 ▸, 2003 ▸).

*PDBeFold* (Krissinel & Henrick, 2004 ▸) analysis using the default threshold of 70% identified the nearest structural neighbor of *Hp*BPL to be the structure of *Mycobacterium tuberculosis* biotin protein ligase (*Mt*BPL) with a nucleoside-based bisubstrate adenylation inhibitor (PDB entry 4xu1; Bockman *et al.*, 2015 ▸). Nucleoside-based bisubstrate adenylation inhibitors of *Mt*BPL have been developed to block the catalytic activity of *Mt*BPL (Bockman *et al.*, 2015 ▸). *Mt*BPL (PDB entry 4xu1) and *Hp*BPL align well and share a well conserved core domain (Fig. 3 ▸*c*). Additional results from *PDBeFold* are detailed in Supplementary Table S1. *Mt*BPL shares less than 22% sequence identity with *Hp*BPL and has been investigated for drug discovery (Duckworth *et al.*, 2011 ▸; Gupta *et al.*, 2010 ▸; Ma *et al.*, 2014 ▸; Bockman *et al.*, 2015 ▸). Structure-based sequence alignment reveals that *Mt*BPL has a more extended N-terminus than *Hp*BPL, while the core structures are well conserved (Fig. 4 ▸). The catalytic lysine Lys110 in *Hp*BPL is conserved and aligns well with its counterpart Lys138 in *Mt*BPL (Figs. 4 ▸, 5 ▸ and 6 ▸).

**Figure 5:**
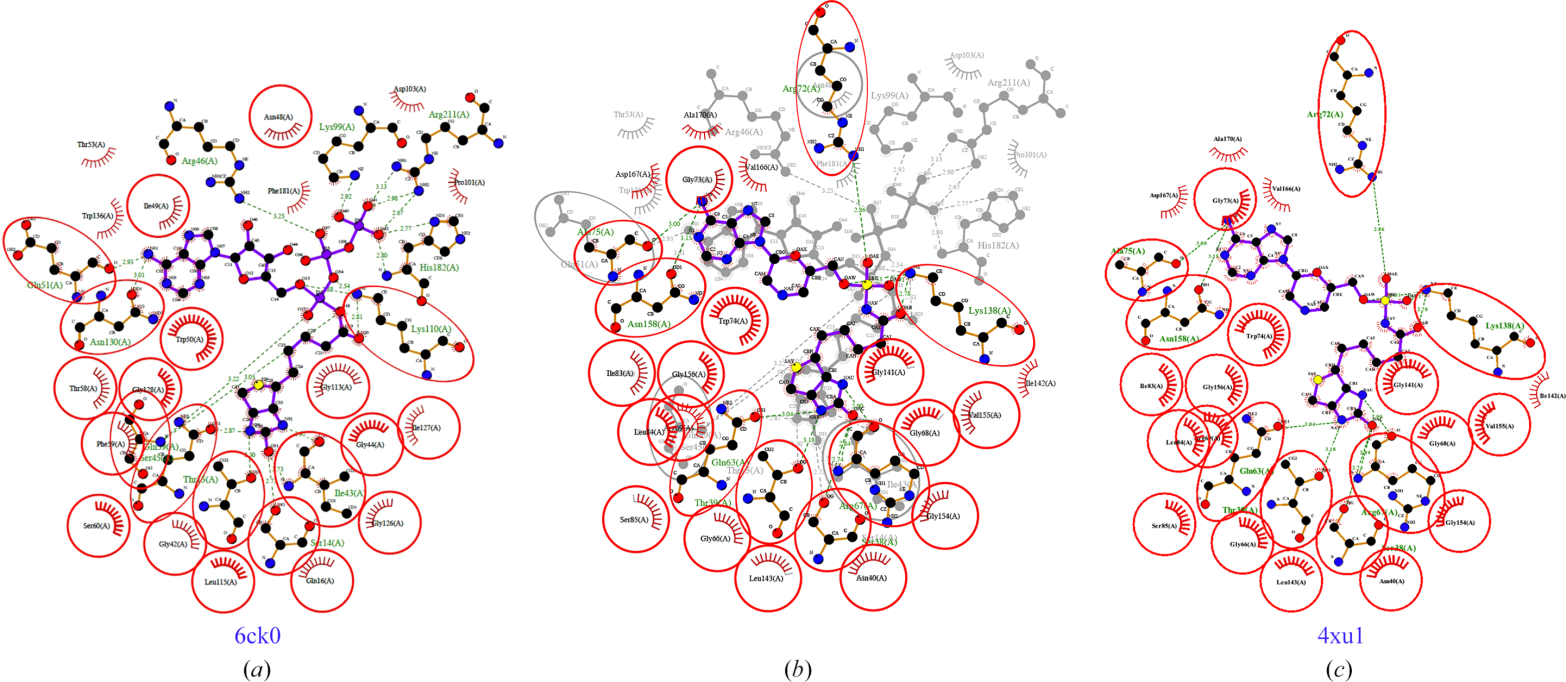
*LigPlus*-generated interaction plots show conserved catalytic cavity residues. Structures are shown of (*a*) *Hp*BPL with biotinyl-5-ATP (PDB entry 6ck0), (*b*) *Hp*BPL with biotinyl-5-ATP (PDB entry 6ck0) superposed with *Mt*BPL with a nucleoside-based bisubstrate adenylation inhibitor (PDB entry 4xu1) and (*c*) *Mt*BPL with a nucleoside-based bisubstrate adenylation inhibitor (PDB entry 4xu1).

**Figure 6:**
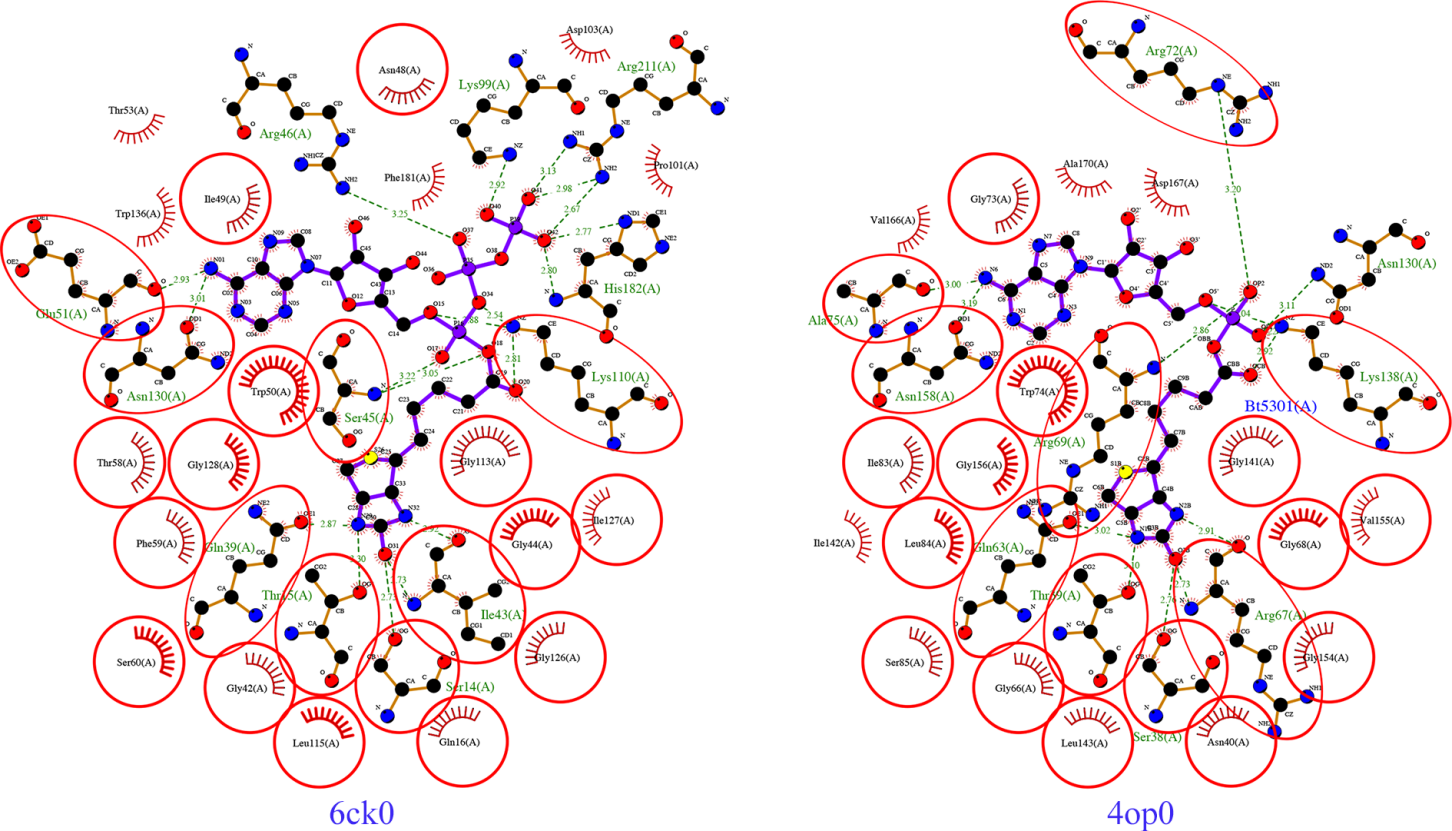
Comparison of biotinyl-5-ATP binding and biotinyl-5-AMP binding by *Hp*BPL (PDB entry 6ck0) and *Mt*BPL (PDB entry 4op0).

Additionally, the active site of *Hp*BPL aligns well with that of *Mt*BPL and appears to be capable of binding the nucleoside-based bisubstrate adenylation inhibitor (Fig. 5 ▸). There is no reported structure of *Mt*BPL with biotinyl-5-ATP, but there is a reported structure with biotinyl-5-AMP (PDB entry 4op0). The active-site residues in the co-crystal structure of *Mt*BPL with biotinyl-5-AMP are well conserved compared with *Hp*BPL, as indicated by the circled conserved residues in a *LigPlus*-generated interaction (Fig. 6 ▸). The pyrophosphate group from biotinyl-5-ATP in our *Hp*BPL structure forms hydrogen bonds with the three catalytic site residues (Arg46, Lys99 and His182). Overall, *Hp*BPL shares significant structural similarities with *Mt*BPL, which is promising for repurposing *Mt*BPL inhibitors. Future studies include a more detailed analysis of *Mt*BPL inhibitors to select those that can be repurposed as *Hp*BPL inhibitors.

## 4. Conclusion

The production, crystallization and 2.25 Å resolution structure of *Hp*BPL reveal a well conserved catalytic cavity and structural similarity to *M*tBPL. Thus, nucleoside-based bisubstrate adenylation and other *M*tBPL inhibitors may be suitable starting points for *Hp*BPL inhibitors.

## Supplementary Material

PDB reference: *Helicobacter pylori* biotin protein ligase, complex with biotinyl-5-ATP, 6ck0

Supplementary Figure and Table. DOI: 10.1107/S2053230X24012056/ir5037sup1.pdf

## Figures and Tables

**Table 1:** Macromolecule-production information

Source organism	*Helicobacter pylori* (strain G27)
DNA source	Nina Salama, Fred Hutchinson Cancer Research Center
Forward primer	5′-CTCACCACCACCACCACCATATGAGACAATGTGAAAAAAGAGTTTTT-3′
Reverse primer	5′-ATCCTATCTTACTCACTTACATCCTATCATAAATCTTACCTTTAAT-3′
Expression vector	BG1861
Expression host	*Escherichia coli* BL21(DE3)R3 Rosetta
Complete amino-acid sequence of the construct produced†	**MAHHHHHH**MRQCEKRVFDSLPSTQTYLLEKLKNNELKAPILIVAKNQSTGIGSRGNIWEGTKSALTFSLALNASDLPKDLPMQANALYLGFLFKEVLKELGSQTWLKWPNDLYLGDQKIGGVLVNVYKGMRVCGIGVNRVSKKWACLDIGASDDLIIEGFLKKIEENLFWGEVLSKYALEFHRSNSFSFHNDWGELVSLKDAELLEDGRICIKGKIYDRM

† The additional N-terminal amino-acid residues are in bold.

**Table 2:** Crystallization

Method	Vapor diffusion, sitting drop
Plate type	Tray 101-d6, 96-well plates
Temperature (K)	290
Protein concentration (mg ml^−1^)	23.6
Ligand mixture composition	6 m*M* MgCl_2_, 6 m*M* ATP, 6 m*M* biotin
Buffer composition of protein solution	20 m*M* HEPES pH 7.0, 300 m*M* NaCl, 5%(*v*/*v*) glycerol, 1 m*M* TCEP
Composition of reservoir solution	0.1 *M* sodium citrate tribasic–citric acid pH 4.0, 0.8 *M* ammonium sulfate (JCSG+ condition B1)
Volume (µl)	0.4
Ratio of drop	1:1
Volume of reservoir (µl)	80
Composition of cryoprotectant solution	0.075 *M* sodium citrate tribasic–citric acid pH 4.0, 0.6 *M* ammonium sulfate, 25%(*v*/*v*) ethylene glycol

**Table 3:** Data collection and processing Values in parentheses are for the outer shell.

Diffraction source	APS beamline 21-ID-F
Temperature (K)	100
Detector	MAR Mosaic 300 mm CCD
Wavelength (Å)	0.97872
Detector distance (mm)	300
Oscillation angle (°)	1
Total No. of frames	360
α, β, γ (°)	122.1, 94.7, 107.9
Resolution range (Å)	47.95–2.25 (2.31–2.25)
Total No. of reflections	93167 (6432)
No. of unique reflections	23767 (1625)
Completeness (%)	97.1 (89.4)
Multiplicity	5.8 (6.2)
〈*I*/σ(*I*)〉	3.9 (4.0)
*R*_r.i.m._	0.055 (0.523)
CC_1/2_ (%)	99.9 (93.7)
Overall *B* factor from Wilson plot (Å^2^)	36.4

**Table 4:** Structure solution and refinement Values in parentheses are for the outer shell.

Resolution range (Å)	47.95–2.25 (2.31–2.25)
Completeness (%)	97.7 (89.4)
σ Cutoff	*F* > 1.97σ(*F*)
No. of reflections, working set	23747 (1428)
No. of reflections, test set	1978 (132)
Final *R*_cryst_	0.171 (0.370)
Final *R*_free_	0.221 (0.430)
No. of non-H atoms	
Protein	3229
Ion	20
Ligand	108
Solvent	116
Total	3473
R.m.s. deviations	
Bond lengths (Å)	0.003
Angles (°)	0.502
Average *B* factors (Å^2^)	
Protein	41.9
Ion	84.4
Ligand	51.6
Water	45.0
Ramachandran plot	
Most favored (%)	97
Allowed (%)	2
Outliers (%)	1
