# Supplementary material for: Co-crystal structure of Helicobacter pylori biotin protein ligase with biotinyl-5-ATP
Source: Acta Crystallogr F Struct Biol Commun. 2025 Jan 1;81(Pt 1):11–8. doi: 10.1107/S2053230X24012056 (PMC11701928; doi:10.1107/S2053230X24012056)
Supplement: Supplementary file 1 [file f-81-00011-sup1.pdf]

STRUCTURAL BIOLOGY
COMMUNICATIONS

Volume 81 (2025)

Supporting information for article:

Co-crystal structure of *Helicobacter pylori* biotin acetyl-CoA carboxylase synthetase (biotin protein ligase) with biotinyl-5-ATP

Jesuferanmi P. Ayanlade, Dylan E. Davis, Sandhya Subramanian, David Dranow, Donald D. Lorimer, Brad Hammerson, Peter J. Myler and Oluwatoyin A. Asojo

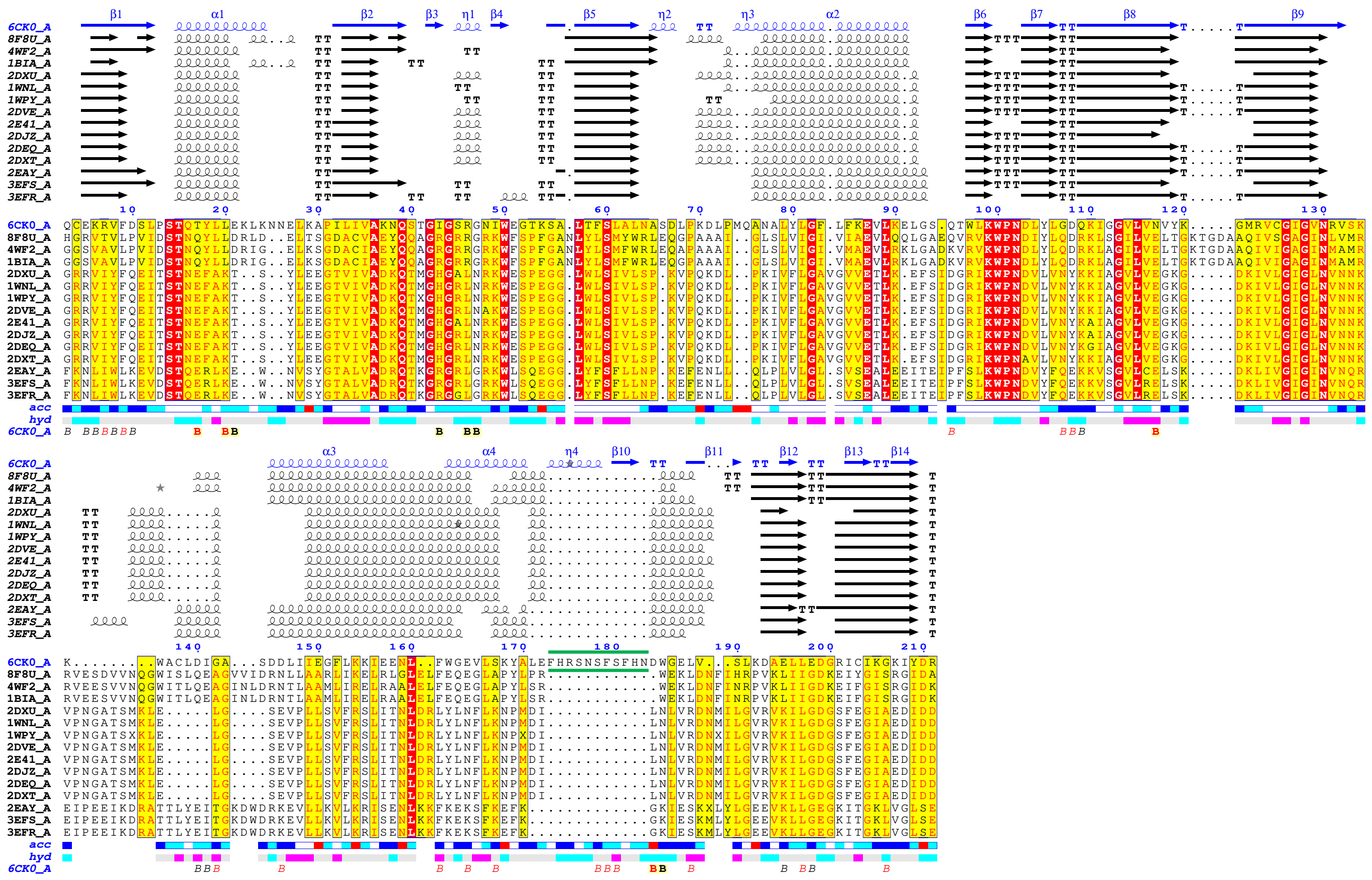

Figure S1 . ENDScript analysis reveals the nearest structural neighbors of *Hp*BPL. The additional amino acid insertion (F173 to N183) is underlined in green. Identical and conserved residues are highlighted in red and yellow, respectively. The different secondary structure elements shown are alpha helices (α), 310-helices (η), beta strands (β), and beta turns (TT).

Table S1 PDBe Fold v2.59. (src3) 14 Apr 2014 result file.

RESULT SUMMARY

##	Q-score	P-score	Z-score	RMSD	Nalgn	Nsse	Ngaps	Seq-%	Nmd	Nres-Q	Nsse-Q	Nres-T	Nsse-T	Query	Target
1	1	66.84	24.62	0.000	210	16	0	1	0	210	16	210	16	PDB 6ck0:B	PDB 6ck0:B
2	0.9564	37.13	18.38	0.314	206	15	1	1	0	210	16	209	17	PDB 6ck0:B	PDB 6ck0:A
3	0.4503	13.27	11.1	1.947	188	14	12	0.2021	0	210	16	263	20	PDB 6ck0:B	PDB 4xu1:A
4	0.4472	10.27	9.872	2.016	189	14	12	0.2011	0	210	16	262	19	PDB 6ck0:B	PDB 4xu2:A
5	0.446	9.458	9.699	2.269	186	14	11	0.2312	0	210	16	235	17	PDB 6ck0:B	PDB 2dkg:A
6	0.4438	7.654	8.893	2.250	185	14	11	0.2324	0	210	16	235	17	PDB 6ck0:B	PDB 1wpy:A
7	0.4438	12.82	10.9	1.970	188	14	12	0.2074	0	210	16	265	19	PDB 6ck0:B	PDB 4xu0:A
8	0.4438	7.668	8.893	2.284	186	14	11	0.2312	0	210	16	235	17	PDB 6ck0:B	PDB 1wnl:A
9	0.4432	7.377	8.762	2.288	186	14	11	0.2312	0	210	16	235	17	PDB 6ck0:B	PDB 1x01:A
10	0.4427	10.28	9.872	1.965	188	14	12	0.2021	0	210	16	266	19	PDB 6ck0:B	PDB 4xtw:A
11	0.4422	9.831	9.85	2.295	186	14	11	0.2312	0	210	16	235	17	PDB 6ck0:B	PDB 1wqw:A
12	0.4418	9.8	9.85	2.265	185	13	12	0.2324	0	210	16	235	17	PDB 6ck0:B	PDB 2dti:A
13	0.4417	7.073	8.608	2.265	185	14	12	0.227	0	210	16	235	18	PDB 6ck0:B	PDB 2zgw:A
14	0.4413	11.82	10.24	2.268	185	14	11	0.227	0	210	16	235	16	PDB 6ck0:B	PDB 2dz9:B
15	0.4407	12.31	10.88	2.005	188	14	12	0.2021	0	210	16	264	20	PDB 6ck0:B	PDB 4xtv:A
16	0.4407	10.13	9.807	1.983	187	14	13	0.2032	0	210	16	263	19	PDB 6ck0:B	PDB 4xu3:A
17	0.4398	9.432	9.547	1.952	187	14	13	0.2032	0	210	16	266	19	PDB 6ck0:B	PDB 4xtx:A
18	0.438	12.88	10.92	2.000	188	14	12	0.2021	0	210	16	266	19	PDB 6ck0:B	PDB 4xtu:A
19	0.4376	10.43	9.958	2.004	188	14	12	0.2021	0	210	16	266	20	PDB 6ck0:B	PDB 4xtz:A
20	0.4371	12.98	10.97	2.019	188	14	12	0.2074	0	210	16	265	19	PDB 6ck0:B	PDB 3rux:A
21	0.4365	10.08	9.785	2.336	186	13	12	0.2258	0	210	16	235	18	PDB 6ck0:B	PDB 2dth:B
22	0.4352	7.566	8.849	2.278	184	14	12	0.2283	0	210	16	235	18	PDB 6ck0:B	PDB 2hni:B
23	0.4343	12.71	10.88	1.947	185	14	12	0.2	0	210	16	264	20	PDB 6ck0:B	PDB 4xty:A
24	0.4328	7.756	8.937	2.329	185	14	11	0.227	0	210	16	235	18	PDB 6ck0:B	PDB 2e1h:B
25	0.4328	7.822	8.959	2.295	184	14	12	0.2337	0	210	16	235	17	PDB 6ck0:B	PDB 1wnl:B
26	0.43	9.61	9.764	2.247	182	13	12	0.2363	0	210	16	235	17	PDB 6ck0:B	PDB 1wpy:B
27	0.4278	10.97	10.15	2.228	181	13	13	0.2376	0	210	16	235	18	PDB 6ck0:B	PDB 2fyk:B
28	0.4269	7.479	8.805	2.304	183	14	12	0.2295	0	210	16	235	18	PDB 6ck0:B	PDB 2e64:B
29	0.4248	9.495	9.693	2.262	179	13	13	0.2346	0	210	16	229	18	PDB 6ck0:B	PDB 2djz:B
30	0.4228	10.87	10.17	2.174	173	12	13	0.2486	0	210	16	221	15	PDB 6ck0:B	PDB 2dxu:B
31	0.4226	10.13	10.35	2.235	177	15	12	0.2373	0	210	16	227	17	PDB 6ck0:B	PDB 1wq7:B
32	0.421	6.768	8.475	2.345	181	13	11	0.2265	0	210	16	230	17	PDB 6ck0:B	PDB 2hni:A
33	0.4195	8.412	8.751	2.330	181	13	13	0.232	0	210	16	232	16	PDB 6ck0:B	PDB 2e41:B
34	0.417	9.325	9.46	2.057	184	14	12	0.2011	0	210	16	263	20	PDB 6ck0:B	PDB 4xtx:B
35	0.4167	12.03	10.6	2.028	181	14	12	0.2099	0	210	16	257	19	PDB 6ck0:B	PDB 4op0:B
36	0.4163	6.674	8.413	2.313	181	13	12	0.232	0	210	16	235	18	PDB 6ck0:B	PDB 2deq:A
37	0.4154	6.642	8.413	2.320	181	13	13	0.2431	0	210	16	235	18	PDB 6ck0:B	PDB 2dto:A
38	0.4154	6.531	8.328	2.320	181	13	13	0.2376	0	210	16	235	18	PDB 6ck0:B	PDB 2dxt:A
39	0.4146	8.359	9.213	2.360	182	13	12	0.2418	0	210	16	235	17	PDB 6ck0:B	PDB 2dth:A
40	0.4137	7.478	8.793	2.298	180	13	13	0.2389	0	210	16	235	17	PDB 6ck0:B	PDB 2dti:B
41	0.4136	6.296	8.221	2.334	181	13	12	0.2376	0	210	16	235	18	PDB 6ck0:B	PDB 2fyk:A
42	0.4134	12.45	10.77	2.028	181	14	12	0.2044	0	210	16	259	20	PDB 6ck0:B	PDB 4op0:A
43	0.4131	7.872	8.982	2.268	179	13	13	0.2402	0	210	16	235	17	PDB 6ck0:B	PDB 2dkg:B
44	0.4128	6.418	8.306	2.340	181	13	12	0.232	0	210	16	235	18	PDB 6ck0:B	PDB 2e41:A
45	0.4111	6.571	8.349	2.318	180	13	13	0.2389	0	210	16	235	18	PDB 6ck0:B	PDB 2dve:A
46	0.4095	6.379	8.264	2.330	180	12	12	0.2389	0	210	16	235	17	PDB 6ck0:B	PDB 1x01:B
47	0.4084	7.779	8.476	2.283	178	13	13	0.236	0	210	16	234	17	PDB 6ck0:B	PDB 2deq:B
48	0.408	5.224	7.718	2.482	183	14	16	0.2787	0	210	16	232	17	PDB 6ck0:B	PDB 3efs:B
49	0.4047	5.209	7.914	2.493	179	12	11	0.2402	0	210	16	223	15	PDB 6ck0:B	PDB 2ejg:B
50	0.4041	9.236	9.589	2.267	177	13	13	0.2373	0	210	16	235	17	PDB 6ck0:B	PDB 2dxt:B
51	0.4021	6.102	8.143	2.331	173	14	16	0.2717	0	210	16	221	17	PDB 6ck0:B	PDB 3fjp:B
52	0.4012	6.618	8.409	2.395	175	14	15	0.2686	0	210	16	222	17	PDB 6ck0:B	PDB 2eay:B
53	0.401	6.827	8.476	2.339	178	13	14	0.2416	0	210	16	234	17	PDB 6ck0:B	PDB 2zgw:B
54	0.3998	6.781	8.475	2.335	173	13	12	0.2312	0	210	16	222	17	PDB 6ck0:B	PDB 2dzc:A
55	0.399	8.434	9.234	2.341	178	13	13	0.2416	0	210	16	235	18	PDB 6ck0:B	PDB 2dz9:A
56	0.3983	4.415	7.62	2.487	179	12	12	0.2402	0	210	16	227	15	PDB 6ck0:B	PDB 2ejf:B
57	0.3982	7.14	9.192	2.421	179	14	14	0.2793	0	210	16	232	18	PDB 6ck0:B	PDB 3efs:A
58	0.3977	9.665	9.589	2.281	176	12	14	0.2443	0	210	16	235	17	PDB 6ck0:B	PDB 2dve:B
59	0.3976	9.563	9.547	2.282	176	12	14	0.2443	0	210	16	235	17	PDB 6ck0:B	PDB 1wqw:B
60	0.3963	6.908	8.541	2.361	171	13	11	0.2398	0	210	16	217	17	PDB 6ck0:B	PDB 2e10:A
61	0.3948	6.296	8.221	2.427	178	14	14	0.2472	0	210	16	231	17	PDB 6ck0:B	PDB 2dxu:A
62	0.3946	5.878	8.054	2.571	183	14	15	0.2678	0	210	16	233	18	PDB 6ck0:B	PDB 3efr:A
63	0.3915	7.944	9.751	2.154	170	12	11	0.2529	0	210	16	232	17	PDB 6ck0:B	PDB 2ejg:A
64	0.3896	8.799	9.401	2.390	175	13	13	0.2229	0	210	16	229	17	PDB 6ck0:B	PDB 1wq7:A
65	0.3856	11.79	10.46	2.156	178	13	13	0.2022	0	210	16	258	18	PDB 6ck0:B	PDB 4xu2:B
66	0.3841	6.674	8.413	2.354	172	13	13	0.2326	0	210	16	227	17	PDB 6ck0:B	PDB 2e65:A
67	0.3832	5.753	7.965	2.492	178	14	13	0.2753	0	210	16	233	20	PDB 6ck0:B	PDB 3efr:B
68	0.3812	7.302	8.541	2.416	170	13	13	0.2647	0	210	16	219	17	PDB 6ck0:B	PDB 2eay:A
69	0.3809	6.005	8.072	2.429	173	13	14	0.2312	0	210	16	226	17	PDB 6ck0:B	PDB 2e64:A
70	0.3798	9.179	9.359	2.191	179	13	16	0.2123	0	210	16	262	18	PDB 6ck0:B	PDB 4xu3:B
71	0.378	6.634	8.391	2.472	172	13	12	0.2267	0	210	16	222	17	PDB 6ck0:B	PDB 2e10:B
72	0.3775	6.499	8.328	2.456	173	13	13	0.237	0	210	16	226	17	PDB 6ck0:B	PDB 2dzc:B
73	0.377	9.288	9.464	2.083	176	13	15	0.2045	0	210	16	264	18	PDB 6ck0:B	PDB 3l1a:A
74	0.3746	8.383	9.024	2.361	173	12	12	0.2428	0	210	16	235	17	PDB 6ck0:B	PDB 2ejf:A
75	0.3741	8.56	9.066	2.192	178	13	14	0.2135	0	210	16	263	18	PDB 6ck0:B	PDB 4xu1:B
76	0.3724	5.813	7.986	2.440	171	12	14	0.2339	0	210	16	225	17	PDB 6ck0:B	PDB 2e1h:A
77	0.3694	10.13	9.417	2.289	168	13	15	0.1131	0	210	16	230	17	PDB 6ck0:B	PDB 3bfm:A
78	0.3679	9.499	9.505	2.405	182	13	17	0.2033	0	210	16	261	18	PDB 6ck0:B	PDB 4xtz:B
79	0.3665	7.377	8.143	2.427	167	13	15	0.2695	0	210	16	219	16	PDB 6ck0:B	PDB 3fjp:A
80	0.3649	9.394	9.443	2.454	182	13	18	0.2033	0	210	16	259	18	PDB 6ck0:B	PDB 4xtw:B
81	0.3609	7.819	8.793	2.313	171	13	15	0.2164	0	210	16	242	18	PDB 6ck0:B	PDB 2cgh:B
82	0.359	11.78	10.46	2.226	175	13	15	0.1943	0	210	16	262	18	PDB 6ck0:B	PDB 4xu0:B
83	0.3483	7.638	8.709	2.410	171	13	16	0.2047	0	210	16	243	18	PDB 6ck0:B	PDB 3l2z:B
84	0.3287	7.254	8.54	2.588	170	13	17	0.2059	0	210	16	240	17	PDB 6ck0:B	PDB 3l2z:A
85	0.3265	7.551	8.666	2.339	162	13	14	0.2099	0	210	16	238	18	PDB 6ck0:B	PDB 2cgh:A
86	0.3248	6.882	8.285	2.373	159	13	14	0.2201	0	210	16	228	16	PDB 6ck0:B	PDB 3l1a:B
